# Postoperative Medial Plantar and Sural Neuropathy With Complex Regional Pain Syndrome

**DOI:** 10.7759/cureus.62017

**Published:** 2024-06-09

**Authors:** Michelle Shin, Selcen Senol, Steven L Gershon

**Affiliations:** 1 Physical Medicine and Rehabilitation, Eastern Virginia Medical School, Norfolk, USA; 2 Pain Medicine, Gershon Pain Specialists, Virginia Beach, USA

**Keywords:** calcaneal fracture, budapest criteria, coexistent neuropathy, post-operative nerve injury, peripheral neuropathy, complex regional pain syndrome, sural neuropathy, medial plantar neuropathy

## Abstract

This case illustrates a distinct presentation of coexistent medial plantar and sural neuropathy leading to the development of complex regional pain syndrome (CRPS) in a 49-year-old male patient. CRPS is a broad medical diagnosis describing prolonged and excessive pain that is out of proportion to exam and has historically been diagnosed according to the Budapest criteria. To our knowledge, this is a rare report of a case of medial plantar and sural neuropathy further complicated with CRPS, status-post calcaneal fracture, surgery, and post-surgical boot placement. The case highlights the complexity of diagnosing and managing multiple concurrent neuropathies and underscores the need for interdisciplinary approaches in treating CRPS to improve patient outcomes.

## Introduction

Medial plantar neuropathy (MPN) is caused by compression of the medial nerve, commonly associated with tarsal tunnel syndrome. MPN can occur either proximally (within the tarsal tunnel) or distally beneath the plantar arch between the navicular tuberosity and abductor hallucis. Repetitive microtrauma to the nerve may come in the form of space-occupying lesions such as ganglion cyst, tenosynovitis of the flexor hallucis longus and flexor digitorum longus tendons, or prior history of surgery [1].

The sural nerve travels inferiorly along the lateral aspect of the Achilles tendon, posterior to the lateral malleolus, and along the lateral foot. It provides cutaneous innervation to the lateral lower third of the leg as well as the dorsolateral aspect of the foot before terminating on the lateral aspect of the fifth toe. Sural neuropathy caused by ankle fracture, repetitive or prolonged external ankle compression, or iatrogenic injury has been most reported in the literature [2].

Complex regional pain syndrome (CRPS) is a broad medical diagnosis describing prolonged and excessive pain that is out of proportion to physical examination [3]. CRPS is a chronic condition characterized by sensorimotor and autonomic symptoms that often follow events such as fractures or procedures. CRPS has historically been diagnosed according to the Budapest criteria [4], which requires that patients report continuous disproportionate limb pain and at least one symptom in three of four symptom groups (i.e. sensory, vasomotor, sudomotor/edema, and motor/trophic) and show at least one sign in two or more of these symptom groups at the time of clinical evaluation. Updating these diagnostic criteria has largely improved both the sensitivity and specificity with which CRPS is diagnosed in clinical settings [5].

Two subtypes of CRPS are commonly recognized. Type I CRPS, also known as reflex sympathetic dystrophy, has an absence of peripheral nerve injury. Type I CRPS represents as much as approximately 90% of clinical presentations diagnosed with CRPS. Type II CRPS, formerly termed “causalgia,” refers to cases in which there is evidence of peripheral nerve injury. Type II CRPS is often confirmed by nerve conduction studies or surgical inspection [6].

## Case presentation

A 49-year-old male patient presented to the pain clinic in May, 2023, with the complaint of persistent left foot pain and neuropathy for two years status post calcaneal fracture repair. After sustaining a fall in July, 2021, while abroad, the patient had suffered a closed fracture of the left calcaneus. Upon returning home, he chose to proceed with surgical intervention and underwent left calcaneal fracture fixation using a headless compression screw. The operation was completed without complication, and the patient tolerated the procedure well. However, six weeks postoperatively, the patient reported that he began to develop severe pain in the left foot after his sutures were removed. He reported some swelling in his cast, and though the pain regressed, he began to develop severe tingling and numbness in various areas of the left foot. He elected to undergo left deep fibular nerve release, which resolved some of his lower leg symptoms.

One year later, the patient returned to the pain clinic due to persisting symptoms. The patient was evaluated with electromyography (EMG) for assessment of left lower extremity neuropathy versus radiculopathy. EMG testing (Tables 1-2) revealed paradoxically larger amplitude in the left lateral plantar nerve compared to the medial plantar nerve. Left sural sensory response demonstrated markedly diminished responses. There was abnormal spontaneous activity at rest in the left abductor hallucis muscle. Testing revealed evidence for several concomitant processes including isolated left-sided MPN and sural neuropathy. Due to evidence of several concomitant neuropathies, the patient was offered nerve decompression, but he declined. The patient was encouraged to consider treatment possibilities including a spinal cord stimulator, peripheral nerve stimulator, local ketamine infusion therapy, and nerve block. 

**Table 1:** Summary of left lower extremity motor nerve conduction studies. CMAP: compound muscle action potential

Nerve	Onset Latency (ms)	CMAP (mV)
Medial plantar	8.67 (Range: <5.4)	3.81 (Range: >3.5)
Lateral plantar	7.45 (Range: <6.3)	5.07 (Range: >3.0)
Inferior calcaneal	7.22	3.74
Peroneal (ankle)	5.34 (Range: <6.0)	4.70 (Range: >2.0)
Posterior tibial (ankle)	5.34 (Range; <6.0)	10.84 (Range; >3.0)

**Table 2:** Summary of left lower extremity sensory nerve conduction studies. SNAP: sensory nerve action potential

Sensory	Onset Latency (ms)	SNAP ($\mu$V)
Medial calcaneal	1.59	14.67
Superficial peroneal	2.91 (Range: <4.0)	26.61 (Range: >10.0)
Sural	2.81 (Range: <4.0)	4.53 (Range: >10.0)

The patient presented to the pain clinic again six months later in May 2023 (current presentation) with circumferential pain around the left first and second toes extending over the metatarsal. He reported symptoms consistent with Raynaud’s phenomenon including a cold, numb sensation in his left foot and toes. He reported aching, stiffness, and constant discomfort in his left foot to the point where he could only find relief by sizing up two and a half sizes. He wore open-toe sandals to this visit due to severe pain. When prompted for his social history, the patient reported stress as well as difficulty sleeping due to his pain. Physical exam demonstrated subjective numbness throughout the sural nerve distribution with marked allodynia throughout the left lower leg and foot. There was marked edema over the right first to second metatarsals as well as the ankle. No overlying skin changes, atrophy, or erythema were noted.

In line with Budapest criteria, this patient experienced continuous disproportionate limb pain over a nearly two-year period. At the time of evaluation, he exhibited marked allodynia, Raynaud’s phenomenon, and edema which correspond to the sensory, vasomotor, and sudomotor symptom groups respectively. Differential included compression neuropathy versus a mild case of CRPS. However, due to his persistent pain out of proportion, temperature changes, allodynia, and edema as well as evidence of several concomitant processes on EMG, it was much more suggestive of CRPS. Given that the patient was status post calcaneal fracture fixation and was placed in a postoperative boot, his symptoms were consistent with a mild case of type II CRPS, a causalgia likely attributable to postoperative peripheral injury of the medial plantar and sural nerves. The patient has since obtained multiple evaluations from both Podiatry and Orthopedics who have offered treatment of nerve entrapment surgically. However, the patient ultimately agreed to explore spinal cord stimulation and sought additional opinions regarding this mode of treatment with Neurosurgery.

## Discussion

In the foot, the MPN is responsible for both sensation and movement. It supplies feeling to the medial plantar foot, plantar aspect of the first to third toes, and medial half of the fourth toe. It also controls the abductor hallucis, flexor hallucis brevis, flexor digitorum brevis, and the first lumbrical muscles. The medial plantar nerve can be compressed anywhere along its path beyond the tarsal tunnel, leading to a specific and somewhat rare condition known as isolated MPN [7]. Sural neuropathy is also a rare condition, typically secondary to trauma, surgery, or soft tissue scarring anywhere along the leg, ankle, or foot. The most frequently affected compression sites include the lateral border of the ankle, the calcaneus, and the fifth metatarsal. Both MPN and sural neuropathy are best diagnosed using EMG. Our case is unique in that it involves the coexistence of two rare conditions, MPN and sural neuropathy, further complicated by CRPS.

The pathogenesis of CRPS is largely unknown, but proposed mechanisms involve either the peripheral or central nervous system, or both [8]. Dimova et al. investigated the clinical phenotypes of patients with CRPS and attempted to classify patients according to their symptom “clusters” [9]. Clinical examination data was obtained from three independent samples of 444, 391, and 202 patients with CRPS and demonstrated three possible clinical phenotypes: central, peripheral, and peripheral pathophysiology. Furthermore, the etiology of CRPS is often multifactorial, including pro-inflammatory changes, maladaptive changes in pain perception and sensitization, sympathetic overactivity, genetic predispositions, and even autoimmune syndromes. Though there are various mechanisms of the various sensory phenotypes of CRPS, it is highly plausible that CRPS can create susceptibility to secondary nerve damage which might provide an explanation for this patient’s MPN [10]. In a meta-analysis and systematic review by Sobeeh et al., secondary hyperalgesia in locations outside of the originally affected area was found to be indicative of central sensitization characterized by increased response of nociceptive pain-perceiving neurons in the central nervous system to normal or sub-threshold afferent input [8]. In this case, however, both the constellation of symptoms and EMG findings point to confirmed multiple nerve injuries, specifically of the medial plantar and sural nerves. The patient’s chronology of fracture, surgical history, and subsequent boot placement are consistent with nerve injury in CRPS type II that often occurs due to trauma, surgery, or other identifiable causes that directly damage or compress a nerve [11].

## Conclusions

To our knowledge, this is a rare case of medial plantar and sural neuropathy further complicated with CRPS, status-post calcaneal fracture, surgery, and post-surgical boot placement. CRPS remains an area of much further exploration for more specific diagnostic identification, sub-classification, and effective treatment. Patients with CRPS require interdisciplinary teams with diverse and adaptable treatment approaches to maximize patient outcomes in the form of minimizing disability and maximizing quality of life and recovery.
